# The Antihyperglycemic Effect of Crude Liang (*Gnetum gnemon* var. *tenerum*) Leaves Powder on Wistar Rats

**DOI:** 10.1155/2023/5630204

**Published:** 2023-08-31

**Authors:** Anunya Suksanga, Sunisa Siripongvutikorn, Rattana Leelawattana, Chutha T. Yupanqui

**Affiliations:** ^1^Functional Food and Nutrition Program, Faculty of Agro-Industry, Prince of Songkla University, Hat Yai 90110, Songkhla, Thailand; ^2^Center of Excellence in Functional Foods and Gastronomy, Faculty of Agro-Industry, Prince of Songkla University, Hat Yai 90110, Songkhla, Thailand; ^3^Faculty of Medicine, Prince of Songkla University, Hat Yai 90110, Songkhla, Thailand

## Abstract

Chlorophyll and chlorophyllin (CHL) demonstrated antidiabetic activity by inhibiting gluconeogenesis and increasing glucose uptake in rats' muscle cells. Liang leaves contain high amounts of chlorophyll and chlorophyllin and may provide an antidiabetic effect. The antidiabetic activity of chlorophyll and CHL contained in Liang leaves, Cu-chlorophyllin (CCL) Liang leaves treated with CuSO_4_, and untreated crude Liang leaves (CLL) were compared using commercial chlorophyllin (CHL) as a reference. Twelve Wistar male rats were separated into 4 groups (3 rats/group); the first was a normal one (based line group), the second were the diabetic rats treated with CHL, while the third and the fourth were the diabetic rats treated with 0.97 g/kg of CCL and CLL, respectively. Diabetic rats were induced by a high fructose diet, before being taken to administer commercial CHL, CCL, and CLL for 7 days. Nonfasting blood glucose and body weight were checked daily. After euthanasia, organ weight, biochemical, hematological, and histopathological properties were evaluated. CCL treatment showed no antihyperglycemic activity in the rat model but caused some biochemical abnormalities and thrombocytopenia. Commercial CHL gave a higher reduction of nonfasting blood glucose (NFBG) than Liang leaves powder CCL or CLL but also showed some signs of abnormal biochemical parameters. CLL exhibited an antihyperglycemic effect, with higher body weight and increased HDL/LDL ratio and thus could be a promising alternative natural source for diabetes treatment.

## 1. Introduction

Diabetes mellitus (DM) is a serious global health disorder. In 2022, more than 400 million diabetic patients were recorded, and numbers are projected to increase to 783 million by 2045 [1]. Diabetes results in health expenditures of at least USD 966 billion, with increased cost of care management [2]. Metabolic disorders of DM are either inadequate production or impaired utilization of insulin [3]. When not enough insulin is present or when cells cease responding to insulin, the metabolism of carbohydrates, fats, and proteins is disturbed [4]. Most plants contain chlorophyll, a green pigment for photosynthesis [5]. Chlorophyll is the most abundant natural pigment and has been considered as hypoglycemic agent via mechanism of free radicals' inhibition [6]. Chlorophyllin is a semisynthetic substance that is created by replacing the chlorophyll magnesium ion with another metal and removing the phytol tail [7]. Recent studies have shown that both chlorophyllin and metformin (a medication for diabetes) inhibit gluconeogenesis and increase glucose uptake by peripheral muscles [8, 9].

Liang (Thai name) is a native vegetable of southern Thailand [10]. Liang may have high potential to lower blood glucose due to its high protein, fiber, and chlorophyll character [11] as a natural antidiabetic nutrient [12]. However, a comparison of the antidiabetic properties of crude plants containing chlorophyll compared to those with induced chlorophyllin is lacking. Therefore, this study compared the antidiabetic activity of crude Liang leaves, Liang leaves treated with CuSO_4_ (Liang chlorophyllin), and commercial chlorophyllin in a Wistar male rat model.

## 2. Materials and Methods

### 2.1. Liang Leaves Preparation for Chlorophyllin Induction

The species of Liang used in this study was *Gnetum gnemon* var. *tenerum*, with voucher specimen number QBG No. 43621 [13]. Liang leaves in the edible stage were divided into two preparations. Preparation 1 comprised crude Liang leaves powder (CLL) and preparation 2 was Cu-chlorophyllin Liang leaves powder (CCL) treated with CuSO_4_. CLL was prepared by removing the leaves from the petiole and washing with 100 ppm chlorinated solution for 15 minutes as decontamination before drying. CCL was prepared by treatment with CuSO_4_ to obtain Cu-chlorophyllin Liang leaves (CCL). The leaves were tied together with a small brush and the stem was slant cut to allow more contact area to absorb 20% CuSO_4_ solution for 120 minutes at 60°C before washing twice in tap water to remove excess CuSO_4_.

### 2.2. Liang Leaves Powder Preparation

The drying process was performed using a vacuum microwave dryer at 3600 watts for 14 minutes per 0.5 kg leaves to achieve *a*_*w*_  <  0.6. The dried leaves were ground and sieved through 280 mesh size. Liang CCL and CLL powders were analyzed for proximate composition following AOAC 2019 assay [14].

*Experimental Animals.* Twelve male Wistar rats (250–300 g), 5 weeks old, were obtained from Nomura Siam International and checked and housed for 7 days for acclimatization to laboratory conditions. After acclimation, the rats were administered with 30% fructose solution every day for 3 weeks to get diabetic symptoms. Throughout this experiment, the rats were reared at the Animal Laboratory Center, Prince of Songkla University, Thailand, according to the animal welfare care system with temperature 22 ± 3°C, relative humidity 55% ± 10%, light intensity 130–325 lux, lighting schedule 12/12 hours (bright/dark), and given filtered water using a reverse osmosis system. All rats received water ad libitum and a standard laboratory diet.

### 2.3. Ethical Approval

The study was approved by the Institutional Animal Care and Use Committee, Ref. No. 55/2021, Prince of Songkla University.

### 2.4. Induction of Diabetes

A literature review and preliminary tests suggested that a high dose (>50 mg/kg bw) of streptozotocin (STZ) was typically used to induce type 1 diabetic (T1D) rats but not used for insulin resistance induction [15] while using long-term fructose feeding at 3-4 weeks which could develop insulin resistance [16]. Therefore, using fructose with low STZ was an effective way to induce diabetic symptoms. The experimental rats were administered with 30% fructose solution instead of drinking water for 4 weeks and then fasted overnight (10–12 hours) before STZ injection [17]. A single intraperitoneal injection of streptozotocin (STZ) dose 35 mg/kg body weight was prepared in 0.1 M citrate buffer (pH 4.5) [18]. After 48–96 hours of STZ injection, blood was drawn by tail incision after the antiseptic process. Blood glucose level was determined using a glucometer (Bluetooth SIG, Inc., UK). Rats with nonfasting blood glucose levels greater than 250 milligrams per deciliter (NFBG >250 mg/dl) were diagnosed as diabetic [19].

*Experimental Groups.* Normal and diabetic rats (NFBG level >250 mg/dl) were divided into four groups (3 rats/group) as follows.

  Group A: Normal rats receiving distilled water.
  Group B: DM + 100 mg/kg of CHL
  Group C: DM + 0.97 g/kg of CCL
  Group D: DM + 0.97 g/kg of CLL

The rats were fasted overnight and received the designed agents via oral gavage every day for 7 days, with NFBG levels assessed on the 1^st^, 3^rd^, and 7^th^ day to determine antihyperglycemic activity.

### 2.5. Toxicity Study

At the end of the study (7 days), the rats were fasted overnight (16–18 hours) before injection with 120 mg/kg of sodium thiopental. After euthanasia, the heart, kidney, spleen, and liver were collected, rinsed with normal saline, and dried with tissue paper. Organ weight was measured using a precision balance for a 2-digit calculation of organ index as shown in the following equation:

(1) $$\begin{array}{c} Organ\;index=\frac{\left(organ\;weight\times 100\right)}{body\;weight}\text{.} \end{array}$$

### 2.6. Biochemical Analysis

At the end of the study (7 days), blood was collected by cardiac puncture. Serum was prepared to determine the function of the liver (serum glutamate-pyruvate transaminase, SGPT, and serum glutamic oxaloacetic transaminase, SGOT), kidney (blood urea nitrogen, BUN, and creatinine), and lipid profile using a Mindray BS-200 clinical analyzer.

### 2.7. Histopathological Study

Internal organ tissues including the liver and kidneys were separately cut and fixed in 10% formalin for 3 days before being dehydrated. Thereafter, the samples underwent automated tissue processing, starting with paraffin wax embedding, thin sectioning at 5 mm thickness, and hematoxylin and eosin staining. The slides were examined under a light microscope to identify any indications of unusual symptoms.

### 2.8. Hematological Analysis

Blood samples from the rats were collected in tubes containing EDTA for hematological analysis (complete blood count (CBC) including hemoglobin (Hb), hematocrit (Hct), total RBC, mean corpuscular volume (MCV), mean corpuscular hemoglobin (MCH), mean corpuscular hemoglobin concentration (MCHC), platelet count, and white blood cells count (WBC)) by using a Nihon Kohden Celltac Es MEK-7300 automated hematology analyzer.

### 2.9. Statistical Analysis

The Statistical Package for the Social Sciences (SPSS) version 28.0 was used to analyze all data (Chicago, IL, USA) with one-way ANOVA followed by Tukey's post hoc test performed to compare differences between the experimental groups. Results were reported as mean ± SEM and a significant value was set at *P*  <  0.05.

## 3. Results

Table 1 shows the proximate compositions of CCL and CLL. Both Liang leaves samples contained mainly protein and dietary fiber. As expected, CCL had a much higher copper level than CLL (almost 110 times), confirming the success of CuSO_4_ treatment process. Besides Cu content, CuSO_4_ treatment did not change the proximate composition of Liang leaves.

**Figure 1:**
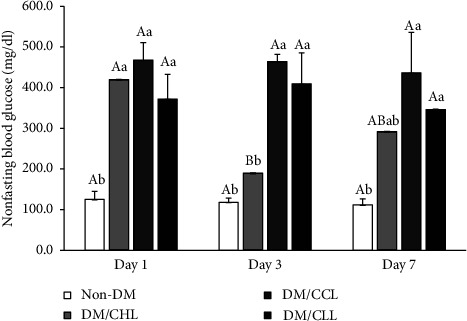
Antihyperglycemic study showing change of NFBG level in screening test study. Different uppercase letters indicate significant differences within a group (*P*  <  0.05). Different lowercase letters indicate significant differences within a day (*P*  <  0.05). Non-DM is the normal rat group; DM/CHL is the diabetic rat group treated with 100 mg/kg of commercial chlorophyllin powder; DM/CCL is the diabetic rat group treated with 0.97 g/kg of Cu-chlorophyllin Liang leaves powder; DM/CLL is the diabetic rat group treated with 0.97 g/kg of crude Liang leaves powder.

The NFBG level of the control or non-DM rats was 125.0 ± 19.9 mg/dl. An increment of NFBG of DM rats treated with CHL, CCL, and CLL gave 419.5 ± 7.2, 467.7 ± 43.1, and 372.0 ± 60.8 mg/dl (*P*  >  0.05), respectively, which was significantly higher than non-DM rats. NFBG of DM rats treated with CCL and CLL reduced, particularly after treatment with CLL but not significantly. Results suggested that using CHL was more effective than using CCL and CLL within 7 days. Extended treatment was hypothesized to be more suitable when using Liang extracts, particularly CLL, which showed a clear trend of reduction in NFBG level (Figure 1).

**Figure 2:**
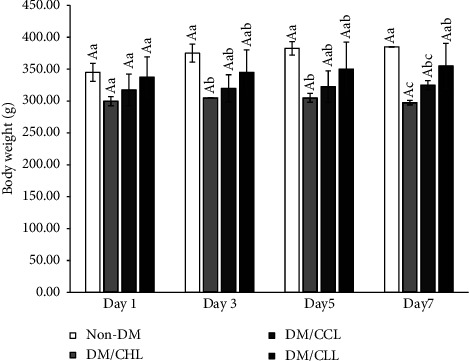
Effects of different treated groups on the body weight of rats. Different uppercase letters indicate mean significant differences within a group. Different lowercase letters indicate significant differences within a day (*P*  <  0.05). Non-DM is the normal rats administered with only distilled water; CHL commercial is the diabetic rats treated with 100 mg/kg of commercial chlorophyllin powder; Liang with CuSO_4_ is the diabetic rats treated with 0.97 g/kg of Cu-chlorophyllin Liang leaves powder; crude Liang is the diabetic rats treated with 0.97 g/kg of crude Liang leaves powder.

Figure 2 shows the effect of CCL, CLL, and CHL on body weight. After treating the rats with CHL, their body weight was lower than the other treatments and lowest on day 7. In general, the body weight of treated rats with CLL recovered faster and was closer to the control group. Rats treated with CLL showed improved body firmness with healthy fur.

No abnormal histological results were found in any key organ in all rat groups. However, the liver, kidney, and spleen of normal rats were smaller than that of the diabetic rats. No differences in organ indices were found among the diabetic groups (Table 2). Slightly larger specific organs were found in DM rats treated with CHL and other Liang powders, possibly due to higher fructose feeding and STZ step effects.

Significant changes were recorded in the liver and kidney function tests (Table 3). Rats treated with CCL had the most elevated transaminase enzymes, blood urea nitrogen (BUN), and creatinine (Cr) levels, while the CHL group showed only higher BUN. The CLL group also had similar levels of transaminase, BUN, and Cr as the normal rat group. Lipid profiles of all diabetic groups showed a pattern of higher HDL, LDL, and triglycerides (TG) than the normal rat group. For DM rats, LDL in all treatments significantly increased by approximately 2 times compared with the non-DM group. HDL in treated DM rats increased but not as much as LDL, with HDL/LDL almost 2 times lower compared with the control group. Results suggested that some compounds may be related to LDL and HDL but further studies are required to confirm this.

**Figure 3:**
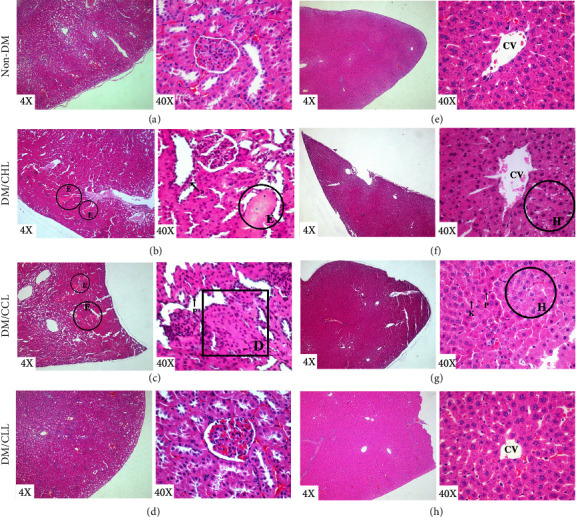
Hematoxylin and eosin staining of kidney (a–d) showing interstitial edema (E), tubular degeneration (D), and pyknosis (P) and liver (e–h) showing hepatocyte hypertrophy (H), karyolysis (K), pyknosis (P), and central vein (CV). Non-DM is the normal rats administered only distilled water; DM/CHL is the diabetic rats treated with 100 mg/kg of commercial chlorophyllin powder; DM/CCL is the diabetic rats treated with 0.97 g/kg of Cu-chlorophyllin Liang leaves powder; DM/CLL is the diabetic rats treated with 0.97 g/kg of crude Liang leaves powder.

After the 7^th^ day, histological studies of the kidney and liver of all animals were conducted, with results shown in Figure 3. Normal rats and diabetic rats receiving CLL showed normal architecture of the kidney and liver. Kidney abnormal architectures were found in diabetic rats treated with CHL and CCL including interstitial edema as a pink area, as shown in Figures 3(b) and 3(c) and pyknosis depicted in Figure 3(b) as a condensed and opaque nucleus. Rats receiving CCL revealed renal tubular cell degeneration (Figure 3(c)) showing cells losing their form and having unclear tubular borders. The CHL and CCL groups encountered problems with their livers as hepatocyte hypertrophy, with the CCL group showing pyknosis and karyolysis (Figures 3(f) and 3(g)) and the total dissolution of chromatin in dying cells.

The hematological indices of all groups are shown in Table 4. CHL rats showed lower WBC, with higher RBC mass presented by higher Hct, more RCB, and higher Hb levels but not MCV, MCHC, and MCH. Interestingly, the CCL group had lower platelet count than the normal, CHL, and CLL groups.

## 4. Discussion

Results confirmed the antihyperglycemic effect of CHL, concurring with Abani et al. [8] who reported that 50 mg/kg BW of CHL effectively reduced blood glucose levels in STZ-induced diabetic mice. Their proposed CHL mechanism for lowering BG was a metformin-like effect wherein it enhances glucose uptake by promoting the expression of GLUT4 (data is in publication process). However, our findings showed that the effect of CHL was rapid but not durable. CHL lowered BG rapidly within 3 days but could not sustain this through 7 days. Based on the NFBG result, CHL treatment was superior to treated CCL and CLL rats. The suspected chlorophyllin contained in CCL was equal to CHL but its action was different, while CLL without chlorophyllin induction demonstrated a marginal antihyperglycemic efficacy. Chlorophyllin (CHL) is used to control blood glucose levels by decreasing gluconeogenesis in hepatocytes [8]. However, it was hypothesized that Cu-chlorophyllin (CCL) in this experiment was too high and harmed hepatocytes and impaired liver function. As a result, CCL was not safe to use to reduce blood glucose levels compared to crude chlorophyll (CLL). Interestingly, results suggested that although the antihyperglycemic activity of CLL was not as rapid as CHL, BG levels of rats receiving Liang products, particularly CLL, reduced while those receiving CHL increased. However, the duration of 7 days may not be long enough to give credence to this efficacy. Furthermore, the antihyperglycemic effects of Liang products may not result from chlorophyllin. Liang leaves contain high fiber and protein, with low carbohydrates as beneficial factors in glucose control [11].

Apart from their marginal effect on blood glucose homeostasis, both CCL and CLL, particularly CLL, could express as protective agents in maintaining body weight. Their high protein content is very important for muscle synthesis, while chlorophyllin in CHL and CCL may have weight-lowering effects since rats treated with CHL had the lowest weight, while CCL groups showed lower weight compared to CLL. This weight-sparing effect of Liang leaves may be due to contained phytochemicals such as phenols and flavonoids [20]. However, no data on body composition of rats were collected at the end of this study to demonstrate changes in specific body composition, especially muscle mass between the groups. Borsheim et al. [21] reported that supplementation of essential amino acids (EAAs) plus arginine improved lean body mass, strength, and physical functions in the glucose-intolerant elderly. Leucine can activate initiation factors involved in protein synthesis [11]; therefore, high levels of leucine contained in Liang leaves may help to increase body weight [21].

Body weight was lower in diabetes-induced rats, with viscera weight remaining in the liver and kidney but not in the heart and spleen, leading to higher organ index values of liver and kidney compared to total body weight. Most studies found lower liver weight and unchanged or lower organ index [22], with higher liver organ index values found in insulin-treated STZ-induced diabetes rats [23], suggesting that the higher liver organ indices in diabetic rats resulted from better insulin action by treatment of CHL, CCL, and CLL. However, insulin action in diabetes on the liver follows glycogen storage which was not tested in the histological study. Fructose intake before STZ is another possibility. Fructose is known to induce fatty liver, and fructose load from diabetic rats could cause fatty liver due to hepatocytes containing more intracellular triglycerides [24]. Unfortunately, the histological study did not show excess triglycerides in the hepatocyte cytoplasm, and the cause of higher organ index values of the liver remains unknown. By contrast, the kidney in early diabetes is well known to be hypertrophy from hyperfiltration due to the osmotic effect of hyperglycemia [25], leading to a heavier kidney.

Changes in lipid profiles in diabetes-induced rats included higher TG, LDL, and HDL. In a hyperglycemic state, the elevation of triglyceride levels triggers an enhancement in the activity of the lipoprotein lipase enzyme [26]. This leads to increase of insulin resistance and decreased in the effectiveness of insulin [27]. With the limitation of lipoprotein lipase enzyme activity, TG lasts longer, leading to high blood levels. The rise in HDL was large, whereas the increment of LDL was smaller. The proportion of elevated LDL was much larger with different baseline levels, leading to reduced HDL/LDL ratios in all diabetic rats, and lowest in CCL; however, the mechanism of this lipid change remains unexplained.

The toxicity of the studied agents in CCL rats suggested significant liver damage by elevation of liver enzymes (SGOT and SGPT). However, the histological study showed significant liver cell hypertrophy in CHL and CCL. Eventual cell death in CCL may be due to damaged cells by high fructose feeding and STZ as the DM induction process. The European Food Safety Authority (EFSA) [28] regulated that specifications for Cu-chlorophyllin must not exceed 8.0% of the total copper chlorophyllin. In this study, CCL was only 1.20% but was suspected to be harmful to the liver and kidney of DM rats. DM induces increased complicity or changes that cause other burdens and diseases.

The histological study showed significant kidney injury in diabetic rats receiving chlorophyllin and both commercial and Cu-chlorophyllin. Kidney injury was found in the histological study, but only mildly elevated BUN and Cr were found in the blood study of the CHL group, with marginal increases in BUN in the CHL group. Intracellular copper was previously reported to lead to kidney fibrosis by stimulating LOX-mediated collagen and elastin crosslinking [29]. The kidney enzyme increased in the early stages and progressed to kidney fibrosis, which is the primary mechanism and the final common route underlying the advancement of all chronic kidney diseases (CKDs) [29]. Copper is a hepatotoxic substance that induces acute or chronic hepatitis by damaging DNA, thereby causing oxidative stress through activation of Kupffer cells [30], [31]. Abnormalities found in the liver also include hepatocyte hypertrophy, karyolysis, and pyknosis which are signs of cell injury and lead to cell death [32, 33]. This finding strongly suggested that toxicity studies should include not only blood but also histological studies. Toxic effects on the liver and kidney were not observed in diabetic rats receiving crude Liang leaves treatment. The hematologic safety study also showed lower WBC in the CHL group, lower platelet counts in CCL, and mild anemia in CLL rats. These hematologic changes suggested significant toxicity of chlorophyllin, both in the commercial and in-house products. Crude Liang leaves caused mild anemia, and this should be monitored further since low WBC and platelet count can lead to severe infection and bleeding.

## 5. Conclusions

Our study showed the efficacy of CLL in lowering blood glucose in diabetic rats with weight maintenance and no toxicity category. The antihyperglycemic effect of CLL was shown to be less and slower than commercial chlorophyllin which can lower blood glucose fast but provide liver, renal, and hematologic toxication. Rats treated with CLL showed more firmness with healthy fur. Further studies on the longer-term effects of Liang leaves are required using a larger number of Wistar rats to draw more accurate conclusions.

## Figures and Tables

**Table 1:** Proximate composition analysis of Cu-chlorophyllin Liang leaves powder (CCL) and crude Liang leaves powder (CLL) (control).

Test list	Unit	Cu-chlorophyllin Liang leaves powder (CCL)	Crude Liang leaves powder (CLL)
Protein	g/100 g	24.38	24.73
Fat	g/100 g	5.39	4.72
Carbohydrate	g/100 g	58.82	56.86
Dietary fiber	g/100 g	40.83	39.27
Soluble dietary fiber	g/100 g	3.01	2.79
Insoluble dietary fiber	g/100 g	37.82	36.48
Ash	g/100 g	6.58	6.53
Copper	mg/kg	1309.44	11.81

**Table 2:** Effect of different treatment groups on organ indices.

Organ	Non-DM	DM/CHL	DM/CCL	DM/CLL
Liver	3.83 ± 0.10^b^	4.22 ± 0.49^a^	4.56 ± 0.17^a^	4.27 ± 0.23^a^
Kidney	0.77 ± 0.03^b^	1.22 ± 0.36^a^	1.15 ± 0.11^a^	1.06 ± 0.08^a^
Spleen	0.21 ± 0.02^b^	0.25 ± 0.01^a^	0.26 ± 0.01^a^	0.25 ± 0.01^a^
Heart	0.32 ± 0.02^a^	0.33 ± 0.04^a^	0.32 ± 0.03^a^	0.33 ± 0.00^a^

Values represent mean ± SEM (*N* = 3). Different lowercase letters indicate significant differences in rows (*P*  <  0.05). Non-DM is the normal rat group; DM/CHL is the diabetic rat group treated with 100 mg/kg of commercial chlorophyllin powder; DM/CCL is the diabetic rat group treated with 0.97 g/kg of Cu-chlorophyllin Liang leaves powder; DM/CLL is the diabetic rat group treated with 0.97 g/kg of crude Liang leaves powder.

**Table 3:** Effect of different treatment groups on biochemical variables of rats.

Parameter	Unit	Non-DM	DM/CHL	DM/CCL	DM/CLL
SGOT	*μ*/L	179.5 ± 62.93^b^	246.50 ± 37.48^b^	1,071.50 ± 28.99^a^	101.50 ± 19.09^b^
SGPT	*μ*/L	46.50 ± 16.26^b^	57.50 ± 3.54^b^	247.50 ± 31.18^a^	48.00 ± 21.21^b^
BUN	mg/dl	20.20 ± 3.96^b^	25.50 ± 1.27^ab^	30.25 ± 0.35^a^	18.20 ± 0.57^b^
Creatinine	mg/dl	0.57 ± 0.14^b^	0.44 ± 0.11^b^	1.09 ± 0.11^a^	0.50 ± 0.01^b^
Cholesterol	mg%	59 ± 9.89^b^	89.5 ± 10.61^a^	74.5 ± 6.36^a^	77 ± 11.31^a^
Triglyceride	mg%	109 ± 62.23^b^	126 ± 11.31^a^	124.5 ± 12.02^a^	124.5 ± 43.13^a^
HDL	mg%	46.5 ± 12.02^b^	64 ± 8.49^a^	53 ± 4.24^a^	56.5 ± 3.54^a^
LDL	mg%	6.5 ± 0.71^b^	13.5 ± 0.71^a^	15 ± 2.83^a^	12.5 ± 3.53^a^
Cholesterol/HDL ratio		1.28 ± 0.12^b^	1.40 ± 0.02^a^	1.41 ± 0.01^a^	1.36 ± 0.12^a^
HDL/LDL ratio		7.3 ± 2.64^a^	4.76 ± 0.88^b^	3.57 ± 0.39^c^	4.67 ± 1.04^b^

Values represent mean ± SEM (*N* = 3). Different lowercase letters indicate significant differences in rows (*P*  <  0.05). Non-DM is the normal rat group; DM/CHL is the diabetic rat group treated with 100 mg/kg of commercial chlorophyllin powder; DM/CCL is the diabetic rat group treated with 0.97 g/kg of Cu-chlorophyllin Liang leaves powder; DM/CLL is the diabetic rat group treated with 0.97 g/kg of crude Liang leaves powder. SGOT is serum glutamic oxaloacetic transaminase; SGPT is serum glutamic pyruvic transaminase; BUN is blood urea nitrogen; HDL is high-density lipoprotein; and LDL is low-density lipoprotein.

**Table 4:** Effect of different treatment groups on rat hematology in the screening test.

Parameter	Unit	Non-DM	DM/CHL	DM/CCL	DM/CLL
WBC	×10^9^/L	4.00 ± 0.84^a^	2.75 ± 0.49^b^	4.00 ± 1.56^a^	4.55 ± 0.07^a^
Total RBC	×10^12^/L	10.03 ± 0.67^ab^	11.75 ± 0.07^a^	9.74 ± 1.22^ab^	8.88 ± 0.09^b^
Hct	%	55.00 ± 4.24^a^	63.50 ± 2.12^a^	53.50 ± 7.78^a^	47.00 ± 1.41^a^
Hb	g/dl	18.50 ± 0.99^ab^	21.50 ± 0.42^a^	18.20 ± 2.26^ab^	16.25 ± 0.35^b^
MCV	Fl	55.00 ± 0.00^a^	54.00 ± 1.41^a^	54.50 ± 0.71^a^	53.00 ± 1.41^a^
MCH	Pg	18.45 ± 0.21^a^	18.30 ± 0.28^a^	18.70 ± 0.00^a^	18.35 ± 0.21^a^
MCHC	%	33.55 ± 0.071^a^	33.35 ± 0.21^a^	33.65 ± 0.07^a^	32.55 ± 0.21^a^
Platelet count	×10^12^/L	875.00 ± 140.71^a^	700.50 ± 77.07^a^	434.50 ± 465.9^b^	744.50 ± 115.25^a^

Values represent mean ± SEM (*N* = 3). Different lowercase letters indicate significant differences in rows (*P*  <  0.05). Non-DM is the normal rat group administered only distilled water; DM/CHL is the diabetic rat group treated with 100 mg/kg of commercial chlorophyllin powder; DM/CCL is the diabetic rat group treated with 0.97 g/kg of Cu-chlorophyllin Liang leaves powder; DM/CLL is the diabetic rat group treated with 0.97 g/kg of crude Liang leaves powder.

## Data Availability

The data supporting the results are included in the manuscript.
